# Disparities in diabetes care: role of the patient's socio-demographic characteristics

**DOI:** 10.1186/1471-2458-10-729

**Published:** 2010-11-25

**Authors:** Rachel Wilf-Miron, Ronit Peled, Einat Yaari, Orna Shem-Tov, Vainer Anna Weinner, Avi Porath, Ehud Kokia

**Affiliations:** 1 Quality Management in Health Care, Maccabi Healthcare Services, Tel-Aviv, Israel; 2 Faculty of Management, Tel-Aviv University, Tel-Aviv, Israel; 3 Faculty of Health Sciences, Ben Gurion University of the Negev, Beer Sheva, Israel; 4 Central Management, Maccabi Healthcare Services, Tel-Aviv, Israel

## Abstract

**Background:**

The commitment to promoting equity in health is derived from the notion that all human beings have the right to the best attainable health. However, disparities in health care are well-documented. The objectives were to explore disparities in diabetes prevalence, care and control among diabetic patients. The study was conducted by Maccabi Healthcare Services (MHS), an Israeli HMO (health care plan).

**Methods:**

Retrospective study. The dependent variables were diabetes prevalence, uptake of follow-up examinations, and disease control. The independent variables were socio-economic rank (SER), ethnicity (Arab vs non Arab), supplementary voluntary health insurance (SVHI), and immigration from Former Soviet Union (FSU) countries. Chi Square and Logistic Regression Models were estimated.

**Results:**

We analyzed 74,953 diabetes patients. Diabetes was more prevalent in males, lower SER patients, Arabs, immigrants and owners of SVHI. Optimal follow up was more frequent among females, lower SERs patients, non Arabs, immigrants and SVHI owners. Patients who were female, had higher SERs, non Arabs, immigrants and SVHI owners achieved better control of the disease. The multivariate analysis revealed significant associations between *optimal follow up *and age, gender (males), SER (Ranks 1-10), Arabs and SVHI (OR 1.02, 0.95, 1.15, 0.85 and 1.31, respectively); *poor diabetes control *(HbA1C > 9 gr%) was significantly associated with age, gender (males), Arabs, immigrants, SER (Ranks1-10) and SVHI (OR 0.96, 1.26, 1.38, 0.72, 1.37 and 0.57, respectively); significant associations with *LDL control *(< 100 gr%) were revealed for age, gender (males) and SVHI (OR 1.02, 1.30 and 1.44, respectively).

**Conclusion:**

Disparities in diabetes prevalence, care and control were revealed according to population sub-group. MHS has recently established a comprehensive strategy and action plan, aimed to reduce disparities among members of low socioeconomic rank and Arab ethnicity, sub-groups identified in our study as being at risk for less favorable health outcomes.

## Background

The commitment to promoting equity in health care is derived from the notion that all human beings have the right to the best attainable health [1]. Adoption of health as a basic human right by the United Nations [2] and by the WHO in 1978 [3] established health equity as a moral commitment for governments and health organizations.

Inequity in health is defined as "the presence of systematic and potentially remediable differences in one or more aspects of health across socially, demographically, or geographically defined populations or population sub-groups" [4]. These differences are not only unnecessary and avoidable, they are considered unfair and unjust [5].

In 2001, the American Institute of Medicine (IOM) suggested six dimensions to define quality of care. One of them is care's equity, meaning that the quality of care is not influenced by personal characteristics such as gender, ethnicity, geography, and social or economic status [6]. However, disparities in health care have been well-documented over the years [7-11].

Israel has a universal health care insurance system that is financed through taxation linked to income and general revenue. Israelis choose between four health plans, referred to in Hebrew as "sick funds". Each non-profit plan is required to provide its members with a basic benefits package that includes physician services, hospitalization and outpatient care. Enrollment in one of the health plans is by free choice; plan denial of enrollment is prohibited.

In addition, all Israeli health plans offer their members the opportunity to purchase supplementary voluntary health insurance (SVHI) that enlarges the package [12]. Despite the universal coverage by a broad package of services, disparities in provision of health services and in health outcomes exist and broadened during the last two decades [13-18].

Diabetes is one of the most common chronic diseases that places a sizeable burden on patients, health care systems, and society [19]. As in other countries, diabetes prevalence in Israel has risen over the last few decades, reaching a prevalence of 6.4% in adults (aged 18+), an annual increase of 0.2% [13].

Maccabi Healthcare Services (MHS) is an Israeli health plan providing community-based health services throughout the country to 1.8 million members from diverse population groups. Services are provided in five regional and 150 local branches (the basic administrative unit), based on a core staff of some 3,000 independent physicians. These physicians are self-employed, working from either MHS clinics located in the branches or their private offices. Most have solo practices. In 2004, MHS implemented an organizational "Performance Measurement System" (PMS) that measures performance according to a set of 24 indicators in six clinical domains, including diabetes. The PMS derives its data from the operational database housed in the organization's main computer. Measures are analyzed and reported on a monthly basis at the regional and branch levels; they are then distributed to all managers and caregivers. These steps cast light on disparities and raised the awareness of equity issues within the organizational discourse. As a result, MHS management has decided to adopt improvement of equity as a strategic goal. One consequence of that decision was to shift analysis of performance data from the organizational unit level (region or branch) to the individual member level in order to better understand the relationship between member characteristics and health outcomes.

This article presents the results of an analysis of the associations between socio-economic status, ethnicity, ownership of supplementary voluntary health insurance(SVHI), immigration, and diabetes prevalence and care measures. In the discussion we describe the strategy and action plan that MHS has taken in wake of the results.

## Methods

### Setting

MHS, the second largest health plan in Israel, with 1,807,521 members when the study was performed.

### Study Period

Data were extracted on November 15, 2008 (Due Day) and reflects care provided during the previous 12 months (December 2007-November 2008)

### Study Subjects

Our reference population for the assessment of *diabetes prevalence *among different groups consisted of all MHS members, aged 18-80, who had visited a general practitioner (GP) at least once during the previous two years. The study population (diabetic patients) for the assessment of *diabetes care and control *were all MHS members, aged 18-80, who were recorded in the computerized Diabetes Registry (DR) on the due day and visited a GP at least once during the previous two years. We used the latter criterion as a proxy for a routine patient-physician relationship in all measures that reflect care at the practice level.

### Diabetes Registry (DR)

Diabetic patients were defined according to the criteria suggested by the American Diabetes Association [20]. Symptoms were diagnosed as diabetes with fasting plasma glucose > 125 mg/dL or casual (namely, any time of day without regard to time since last meal) plasma glucose concentration ≥200 mg/dL, in addition to a diabetes diagnosis documented in the medical records. Patients were included also if they (1) purchased at least two hypoglycemia medications or a single insulin dose during any six-month period or (2) had HbA1C results of at least 7.25 gr%. HbA1C of 6.5 gr% was used as an entry criterion only if a diabetes diagnosis had been documented in the electronic medical records [21]. Women of child-bearing age who entered the DR due to one purchase of insulin but without any other diabetic medications or no purchase of insulin for the next three months were considered Gestational Diabetics and subsequently excluded.

### Data sources

Data were extracted from: (1) the MHS computerized DR and billing system, (2) the MHS computerized PMS, (3) Israel's Census Registry for data on Socio-Economic Rank (SER) and ethnicity.

### Performance measures

In order to assess the effectiveness of management of diabetes care and its potential complications, we analyzed the following quality indicator *process measures*: HbA1C and LDL cholesterol testing, a pre-defined optimal follow-up, and intermediate outcome measures: HbA1C control and LDL cholesterol control.

### Definitions

#### HbA1C testing

Proportion of patients registered in the DR who performed at least one HbA1C test during the previous year.

#### LDL cholesterol test

Proportion of patients registered in the DR who performed at least one LDL cholesterol test during the previous year.

#### Optimal Follow-up

This is an "all or none measure" [22], which includes performance of all the following tests: HbA1C testing, LDL Cholesterol testing, nephropathy monitoring (urine micro-albumin), eye and foot exams, blood pressure and Body Mass Index recording.

### Intermediate outcomes

(1) *Poor HbA1C control: *Proportion of DR patients who performed an HbA1C test during the previous year and had demonstrated HbA1C ≥9 gr%; (2) *Appropriate LDL control*: Proportion of DR patients who performed an LDL test in the previous year and had achieved LDL cholesterol ≤ 100 mg%.

### Socio Economic Rank (SER)

Israel is divided into geographic sub-districts, each with a population of approximately 3,000. Israel's Central Bureau of Statistics characterizes each sub-district according to a socio-economic status ranking system based on the 1995 national census, updated in 2003. Sub-districts are ranked on a scale of 1-20, with 1 being the lowest and 20 the highest. The following variables are among those included in the SER equation: housing density, household employment, income, and education [23]. In our analysis, subjects were assigned to a sub-district according to his or her last recorded address and then ranked for socio-economic status by that sub-district.

### Supplementary Voluntary Health Insurance (SVHI)

As mentioned, Israel has a universal health care system with a unified benefits package of services provided by four health plans. All health plans offer their members the opportunity to purchase SVHI for services that are not included in the basic package, such as certain types of medical devices or greater choice among the services included in the package. Every member, irrespective of health status, can purchase his or her plan's SVHI; the tariff is age-based and not related to health status. Relevant benefits that diabetic patients can utilize include, for example, reduced prices when purchasing devices for self-monitoring of blood glucose levels, reimbursement for consultations with private experts; and reduced co-payment for sophisticated new drugs. 87% of DR MHS members and 88% of all MHS members have SVHI.

### Ethnicity (Arabs vs. Non Arabs)

Since personal data on ethnicity are not available to HMOs, Israeli Arabs were identified by the ethnicity of their residence in a settlement or (in large mixed cities such as Nazareth) by their sub-district, as recorded in the National Census. By using this method, we estimate that 90% of MHS's Arab members were allocated.

### Immigration

Starting in 1990, Israel became the objective of a wave of immigration (about 1 million people) from countries belonging to the Former Soviet Union (FSU). These immigrants contributed to 15% growth in total population. The MHS data set contains immigration status for this group of immigrants only. Thus, under the term "Immigration," we considered only these immigrants. We should note that other immigration groups arriving during this period are relatively small in number.

### Data set construction

We combined the MHS DR, Billing System, PMS, and Israeli Census into one data set using the AS400 query system.

### Statistical Methods

(A) We performed an analysis of variance for prevalence as well as for process and intermediate outcome measures, between sub-groups (Gender, SERs, Ethnicity, Immigration and SVHI) using Chi-square techniques; (B) Logistic Regression Models were estimated for three dependent variables: (1) Optimal follow-up; (2) recorded HbA1C > 9 gr% and (3) achieving LDL < 100 gr.%. The independent variables were included in the models according to whether they reached the significance level (p ≤ 0.05) in the univariant analysis. For SER, we included SER 1-10 and 11-20 as two separate dummy variables in the final models although we compared four ranks in the univariant analysis. Our decision was based on the fact that in the univariant analysis, the SER categories 1-5 and 6-10 differed from the two others (11-15 and 16-20), and the preliminary models indicated robust results only for two rather than four dummies. Odds Ratios (OR) and 95% Confidence Intervals (95%CI) were calculated.

## Results

MHS members aged 18-80 who had visited their GP at least once during the previous two years numbered 1,039,055. Of these, 74,953 diabetic patients were eligible for participation in this study and thus served as our study population. Included were 53.9% males and 46.1% females; mean age was 58.8 (± 11.4) and 60.1 (± 12.2) for males and females, respectively; 6% were ineligible due to the fact that they have not visited their GP at least once during the previous two years.

### Diabetes Prevalence

Diabetes was more prevalent in males when compared to females, members with a lower SERs (1-10) when compared to a higher SERs (11-20), Arabs as opposed to non Arabs, immigrants from FSU countries when compared with veteran residents, and SVHI owners when compared to non owners. All differences were statistically significant (p < 0.001; see Table 1).

**Table 1:** Prevalence (%) and process measures performed (%) by gender, SER, immigration, ethnicity and SVHI

Variable	Prevalence	P Value	HbA1C Testing	P Value	LDL Testing	P Value	Optimal F/U	P Value
**Gender**								
Males	8.4		85.0		84.3		23.7	
**Females**	6.2	< 0.001	86.3	< 0.001	85.2	< 0.001	24.7	0.002
**SER**								
1-5	9.0		86.2		84.2		26.5	
6-10	8.8		86.2		84.9		24.5	
11-15	7.7		85.7		85.2		23.5	
16-20	6.2	< 0.001	85.1	< 0.001	84.7	< 0.001	24.9	< 0.001
**Ethnicity**								
Arabs	8.2		86.3		83.5		21.2	
Non Arabs	7.1	< 0.001	85.6	0.276	84.8	0.049	24.4	< 0.001
**Immigration**								
Immigrants	10.1		87.5		86.7		24.3	
Veterans	6.7	< 0.001	85.0	< 0.001	84.7	< 0.001	24.1	0.580
**SVHI**								
Owners	7.3		86.3		85.3		24.6	
Non Owners	6.8	< 0.001	80.8	< 0.001	80.4	< 0.001	20.3	< 0.001

### HbA1C Testing

Higher percentages of HbA1C testing were observed in females when compared to males, members with a lower SERs (1-10) when comparing to a higher SERs (11-20), and immigrants from FSU countries when compared to veteran residents, and SVHI owners when compared to non owners. All differences were statistically significant (p < 0.001). Differences in performing HbA1C between Arabs and non Arabs were not statistically significant (P = 0.276; see Table 1).

### LDL Testing

Higher percentages of LDL testing were observed for females when compared with males, non Arabs when compared with Arabs, immigrants from FSU countries when compared to veteran residents, and SVHI owners when compared to non owners. All differences were statistically significant (p < 0.001) for Arabs as compared with non Arabs (P = 0.049). Differences in performing LDL testing by SER were not statistically significant (P = 0.152; see Table 1).

### Optimal Follow Up

Higher percentages of optimal follow up were observed for females when compared to males, lower SERs (1-5) when comparing with other SER ranks, non Arabs when compared to Arabs, and SVHI owners when compared with non owners. All differences were statistically significant (p < 0.001; for Arabs when compared with non Arabs, P = 0.049). Differences in performing LDL testing between FSU immigrants and veteran residents were not statistically significant (P = 0.580; see Table 1).

### Intermediate Outcomes - Disease Control

#### Poor diabetes control

The percentage of patients achieving HbA1C levels exceeding 9 gr% was higher for males when compared with females, for patients belonging to the 1-5 and 6-10 SERs when compared with those belonging to the 11-15 and 16-20 SERs, for Arabs when compared with non Arabs, for veterans when compared to immigrants from FSU countries and for non owners when compared to owners of SVHI. All differences were statistically significant (p < 0.001; see Table 2).

**Table 2:** Intermediate outcome measures achieved (%), by gender, SER, immigration, ethnicity and SVHI

Variable	HbA1C > 9 gr%	P Value	LDL < 100	P Value
**Gender**				
Males	10.9		55.5	
Females	8.5	< 0.001	50.0	
**SER**				
1-5	13.9		51.6	
6-10	10.9		51.8	
11-15	8.5		53.3	
16-20	7.3	< 0.001	55.9	
**Ethnicity**				
Arabs	20.5		45.4	
Non Arabs	9.1	< 0.001	53.5	
**Immigration**				
Immigrants	7.2		53.4	
Veterans	10.5	< 0.001	52.9	
**SVHI**				
Owners	9.0		54.0	
Non Owners	15.9	< 0.001	45.0	

#### Appropriate LDL control

The percentage of patients achieving LDL cholesterol levels below 100 gr% were higher among males when compared with females, among members in the 11-15 and 16-20 SERs when compared with those in the 1-5 and 6-10 SERs, among non Arabs when compared with Arabs, and among SVHI owners when compared with non owners. All differences were statistically significant (p < 0.001; see Table 2).

#### Multi-Variant Analysis

The regression models for *optimal follow up *revealed positive and statistically significant associations with age (OR = 1.02 95%, 95%CI = 1.019-1.022); SVHI (OR = 1.31, 95%CI = 1.239-1.400) and SER 1-10 (OR = 1.15, 95%CI = 1.107-1.198). Negative associations were revealed with being Arab (OR = 0.85, 95%CI = 0.766-0.950) and male (OR = 0.95, 95%CI = 0.924-0.995) (see Table 3). For *poor diabetes control*, positive and statistically significant associations were found with being male (OR = 1.26, 95%CI = 1.193-1.346), Arab (OR = 1.38, 95%CI = 1.219-1.581), and having an SER of 1-10 (OR = 1.37, 95%CI = 1.288-1.463). Negative but not significant associations were found with age (OR = 0.96, 95%CI = 0.963-0.968), ownership of SVHI (OR = 0.57; 95%CI = 0.531-0.629) and being an immigrant from the FSU (OR = 0.72, 95%CI = 0.669-0.784) (see Table 4). For *achieving adequate LDL cholesterol control*, positive and significant associations were found with age (OR = 1.02, 95%CI = 1.026-1.029), being male (OR = 1.30, 95%CI = 1.260-1.351), and SVHI ownership (OR = 1.44, CI = 1.366-1.529) (see Table 5).

**Table 3:** Results of the logistic regression analysis: final model for dependent variable: optimal follow up

Variable	B	**SE**^**1**^	P Value	**OR**^**2**^	**95% CI**^**3**^
Age	0.020	0.001	< 0.001	1.02	1.019-1.022
Gender	-0.042	0.019	0.026	0.95	0.924-0.995
Ethnicity	-0.159	0.055	0.004	0.85	0.766-0.950
SER1-10	0.141	0.020	< 0.001	1.15	1.107-1.198
SVHI	0.276	0.031	< 0.001	1.31	1.239-1.400

^1^Standard Error; ^2 ^Odds Ratio; ^3^95% Confidence Interval.

**Table 4:** Results of the logistic regression analysis: final model for dependent variable: HbA1C > 9 gr%

Variable	B	**SE**^**1**^	P Value	**OR**^**2**^	**95% CI**^**3**^
Age	-0.035	0.001	< 0.001	0.96	0.963-0.968
Gender	0.237	0.031	< 0.001	1.26	1.193-1.346
Ethnicity	0.328	0.066	< 0.001	1.38	1.219-1.581
SER1-10	0.317	0.032	< 0.001	1.37	1.288-1.463
SVHI	-0.548	0.043	< 0.001	0.57	0.531-0.629
Immigration	-0.323	0.040	< 0.001	0.72	0.669-0.784

^1^Standard Error; ^2^Odds Ratio; ^3^95% Confidence Interval.

**Table 5:** Results of the logistic regression analysis: final model for dependent variable: LDL < 100 gr%

Variable	B	**SE**^**1**^	P Value	**OR**^**2**^	**95% CI**^**3**^
Age	0.027	0.001	< 0.001	1.02	1.026-1.029
Gender	0.266	0.018	< 0.001	1.30	1.260-1.351
Ethnicity	-0.094	0.050	0.061	0.91	0.826-1.005
SER1-10	-0.002	0.019	0.908	0.99	0.961-1.036
SVHI	0.368	0.29	< 0.001	1.44	1.366-1.529

^1^Standard Error; ^2^Odds Ratio; ^3^95% Confidence Interval .

## Discussion

This study was conducted to identify the associations between socioeconomic and demographic characteristics and quality of diabetes care in order to identify populations at risk for less favorable health outcomes. Our findings indicated differences in diabetes prevalence and effective disease management between population groups as defined by their socioeconomic position and/or ethnicity. These findings are of special importance in light of the fact that Israel has a national health care system providing a universal broad benefit package of services.

### SER

With respect to MHS members, we found a negative association between socioeconomic status and diabetes prevalence as well as intermediate outcome measures. These differences are not new. In 2007 it was published that diabetes prevalence in Israel was five times greater for people with social security tax payment exemptions (a proxy for low socioeconomic ranking) when compared with the general population [13]. Diabetes prevalence was twice as high among Israelis having only eight years of education when compared with those with at least twelve years of education (12% and 5.8%, respectively). Among Arabs, the gaps were even larger (11.5% and 3.7% for 8 and 12+ years of education, respectively) [23]. In a report published in 2007 by the U.S. Agency for Healthcare Research and Quality, disparities in diabetes management and complications among American population sub-groups were demonstrated. White adults with high incomes and some college or higher education were more likely to receive recommended diabetic services and less likely to be exhibit disease-related complications [24].

Outcomes of diabetes care are multifaceted, comprising aspects of patient education regarding the taking of personal responsibility and self-care, compliance with customized treatment plans, maintenance of a strict diet, adoption of a healthy life style, and automatic and regular checking of blood glucose levels. These actions are associated with personal socio-demographic characteristics such as education, income, and mobility, as well as psychological, social, and cultural resources.

Based on extensive recently compiled documentation, it appears that communities in Israel's peripheries suffer from inadequate access to high quality of care [25]. The inhabitants of those regions also tend to be from poor socio-economic backgrounds. We assume that due to the inability of health organizations to mobilize good caregivers willing to serve in these regions, the likelihood that the residents will achieve poor health outcomes is increasing.

Our findings also revealed that members from poor socio-economic backgrounds performed relatively well according to process measures but demonstrate wide disparities regarding intermediate outcome measures when compared with MHS members from better socio-economic backgrounds. These findings are compatible with national studies [13]. We can conclude that it is relatively easy to encourage patients from poor socio-economic backgrounds to perform these tests because they require fewer personal resources than do the achievement of good health outcomes. Research into barriers to favorable outcomes is required in order to develop more appropriate disparity-reducing strategies.

### Ethnicity

Results from the analysis of diabetes prevalence, follow-up examinations and HbA1C control demonstrated considerable disparities between Israel's Arab and non Arab population. These findings further confirm the findings from our previous study, which demonstrated disparities in mammography utilization among MHS Arab members and which suggested that language and socio-cultural barriers contributed to these gaps [26]. Other national-level reports present similar findings [27].

The literature provides significant evidence of the health disparities suffered by ethnic minorities [24]. One interesting finding published recently is that not all of the disparities described can be related to individual characteristics. Evidence of racial differences in diabetes outcomes that were related to physician practice has been found. One study showed that individual physicians achieved less favorable outcomes among their black than among their white patients [28]. These findings challenge society and health organizations to identify solutions for "unequal treatment" [29]. MHS has begun implementing interventions tailor-made for the Arab population [26]; see below for its organization-wide action plan.

### Supplementary Voluntary Health Insurance (SVHI)

As mentioned, MHS offers its members the opportunity to purchase SVHI for services not included in the basic package, such as certain types of medical devices or greater choice among the services included in the package. 87% of MHS members overall, and the same percentage among diabetic patients, have purchased SVHI.

Our findings suggest that MHS members who purchased SVHI are more likely to perform optimal follow up and achieve appropriate disease control. Follow-up examinations, for example, are relatively simple to perform and free of charge - irrespective of SVHI ownership. Therefore, it appears that financial barriers to purchasing SVHI or paying the relatively small co-payment when using the relevant services are probably not the causal agent for less favorable health outcomes between groups; instead, it appears that the lack of SVHI is an important indicator for a variety of population characteristics that are associated with poor health habits and other yet unidentified intervening variables that might contribute to poorer health outcomes. These findings support our previous suggestion regarding the influence of individual resources rather than financial resources on health.

### Immigration

In the 1990s, Israel became home to a huge wave of immigration, primarily from Former Soviet Union countries. This group is characterized by its high educational level, relatively good assimilation into Israeli society and relatively long residence in Israel (10 years and more) at the time of the study. These factors, in addition to other special characteristics of these group, may explain our findings. Cultural and language barriers might also be less significant since many of the GPs and nurses treating MHS patients are themselves former immigrants from the same countries.

### From analysis to an action plan

The results of this study helped our organization to identify members from a poor socioeconomic background and those who are from diverse ethnicity as populations at risk for health disparities. These findings were presented to MHS central management by it's division of quality management  who had previously experienced the ability of local improvement-oriented initiatives to promote care while decreasing disparities [26].

As a result, in 2009 MHS decided to adopt equity, or the reduction in disparities, as a strategic organizational mission. This was translated to an action plan applied in the following major domains: (1) Preferential allocation of resources - a dedicated or "marked" budget - to regional and branch organizational units that serve weak populations; (2) Adaptation of MHS services to the special needs of culturally diverse populations, including recruitment of caregivers and managers from minority communities to better represent these population groups.

It requires long-term perseverance and dedication to reduce disparities. This means going beyond the initial efforts made by MHS and some other Israeli health plans. There is great need for a joint nationwide effort involving various stakeholders - governmental and non-governmental, HMOs, other social and health organizations and patient representatives- to close the existing gap in health outcomes.

## Limitations

### Eligibility

Our subjects were eligible for participation in the study if they had visited their GP at least once during the previous two years. This criterion was set because we wanted our study population to be as homogeneous as possible in service utilization and patient-physician relationship. Six percent of our members were thus excluded from our analysis because they were ineligible. A preliminary analysis of diabetic patients who have not visited their GP in the previous two years indicated that this is a population at great risk for poor health outcomes and should therefore be analyzed separately. We do not consider this fact as a selection bias.

### Ethnicity

Ethnic identity is not available to HMOs for historical reasons, mainly to avoid discrimination. Hence, we identified Arab members by location of residence. We estimate that 90% of this group was identified and participated in our study. We now know that information on ethnicity and other socio-cultural characteristics is crucial to ensure equitable health outcomes. New methods are needed to precisely identify Arab members and to provide them with culturally appropriate, tailor-made services.

### Immigration

In this study we gathered data on immigrants who had arrived to Israel from the FSU. This group comprises the vast majority of recent immigrants living in our country and insured by MHS. Efforts should be made to reach other, samller immigrant groups (e.g., Ethiopians).

### SER

We classified our members according to the SERs assigned to their locality by the Israel Central Bureau of Statistics (CBS) because MHS does not collect individual data on socio-economic status, such as educational level and income. This information is available at the CBS and social security authorities but not transferred to the health plans for regulatory reasons. Although we assumed that districts with populations of at least 3,000 would be homogeneous, direct individual data would be preferable.

### Indirect Evaluation of Outcomes

We were able to analyze only intermediate outcome measures known to be associated with diabetic complications. Our databases are not designed to directly analyze diabetes-related mortality and other diabetes complications, mainly due to the inadequate data flowing from hospitals to the health plans.

## Conclusion

Low socio-economic status and belonging to an ethnic minority are associated with less favorable diabetes care and control. Immigration from FSU countries was not a factor influencing diabetes care and outcomes. Lack of supplementary health insurance emerged as an indicator for a range of population characteristics associated with poor health outcomes. The action plan that MHS has formulated is aimed at allocating managerial, staff and financial resources to populations at risk. We hope that these actions will result in a substantial reduction in disparities over the coming years.

### Ethics

The study did not require an ethical committee approval because the statistical analysis were based on data obtained from anonymous files maintained by MHS. Furthermore, the raw data, containing private confidential information are unavailable to entities outside the MHS management system and require formal approval prior to access. The authors did not require such approval because the data processing and analysis were part of their professional involvement, as MHS staff, to improve the quality and equity of care.

## Competing interests

The authors declare that they have no competing interests.

## Authors' contributions

RWM: Researched the data, wrote the manuscript

RP: Performed the statistical analysis and was involved in writing the manuscript

EY: Researched the data, wrote the manuscript

OST: Involved in data research, reviewed the manuscript

AW: Researched the data

AP: Reviewed the manuscript

EK: Reviewed the manuscript

All authors read and approved the final manuscript.

## Pre-publication history

The pre-publication history for this paper can be accessed here:

http://www.biomedcentral.com/1471-2458/10/729/prepub
